# Enhanced IRE1α Phosphorylation/Oligomerization-Triggered XBP1 Splicing Contributes to Parkin-Mediated Prevention of SH-SY5Y Cell Death under Nitrosative Stress

**DOI:** 10.3390/ijms24032017

**Published:** 2023-01-19

**Authors:** Tsung-Lang Chiu, Hsin-Yi Huang, Hui-Fen Chang, Hsin-Rong Wu, Mei-Jen Wang

**Affiliations:** 1 Division of Neurosurgery, Neuro-Medical Scientific Center, Hualien Tzu Chi Hospital, Buddhist Tzu Chi Medical Foundation, Hualien 970, Taiwan; 2 School of Medicine, Tzu Chi University, Hualien 970, Taiwan; 3 Department of Medical Research, Hualien Tzu Chi Hospital, Buddhist Tzu Chi Medical Foundation, Hualien 970, Taiwan

**Keywords:** parkin, Parkinson’s disease, neuroinflammation, nitric oxide, nitrosative stress, IRE1α, XBP1, oligomerization

## Abstract

Mutations in parkin, a neuroprotective protein, are the predominant cause of autosomal recessive juvenile Parkinson’s disease. Neuroinflammation-derived nitrosative stress has been implicated in the etiology of the chronic neurodegeneration. However, the interactions between genetic predisposition and nitrosative stress contributing to the degeneration of dopaminergic (DA) neurons remain incompletely understood. Here, we used the SH-SY5Y neuroblastoma cells to investigate the function of parkin and its pathogenic mutants in relation to cell survival under nitric oxide (NO) exposure. The results showed that overexpression of wild-type parkin protected SH-SY5Y cells from NO-induced apoptosis in a reactive oxygen species-dependent manner. Under nitrosative stress conditions, parkin selectively upregulated the inositol-requiring enzyme 1α/X-box binding protein 1 (IRE1α/XBP1) signaling axis, an unfolded protein response signal through the sensor IRE1α, which controls the splicing of XBP1 mRNA. Inhibition of XBP1 mRNA splicing either by pharmacologically inhibiting IRE1α endoribonuclease activity or by genetically knocking down XBP1 interfered with the protective activity of parkin. Furthermore, pathogenic parkin mutants with a defective protective capacity showed a lower ability to activate the IRE1α/XBP1 signaling. Finally, we demonstrated that IRE1α activity augmented by parkin was possibly mediated through interacting with IRE1α to regulate its phosphorylation/oligomerization processes, whereas mutant parkin diminished its binding to and activation of IRE1α. Thus, these results support a direct link between the protective activity of parkin and the IRE1α/XBP1 pathway in response to nitrosative stress, and mutant parkin disrupts this function.

## 1. Introduction

Parkinson’s disease (PD) is the second highest cause of age-related neurodegenerative disorder and is characterized by loss of dopaminergic (DA) neurons in the substantia nigra (SN) pars compacta. The majority of PD is apparently sporadic, with only 10% of all cases being familial [1]. Although the processes leading to progression of sporadic PD are not thoroughly understood, clues about the pathogenesis of familial forms of PD have been reinforced by the identification of various gene mutations [2]. Among the genes that are responsible for familial variants of PD, approximately one-half of autosomal recessive juvenile PD (AR-JP) cases result from loss-of-function mutations in the parkin gene (*PARK2*) [3,4]. Parkin, characterized as an E3 ubiquitin ligase and transcription factor [3,5], has been shown to exert neuroprotective effects against several neurotoxic stimuli in vitro and in vivo [6,7,8,9,10].

An increasing body of evidence has indicated that neuroinflammation is implicated in the pathogenesis of PD [11,12]. Microglial activation and accumulation of pro-inflammatory factors, associated with degeneration of DA neurons in the SN, have been observed in various PD animal models and PD patients [13,14,15]. The release of pro-inflammatory and neurotoxic mediators, such as interleukin-1β, tumor necrosis factor-α, nitric oxide (NO) and reactive oxygen species (ROS), from activated microglia is believed to contribute to progressive neuronal damage in neurodegenerative disorders [16,17]. NO is a multifunctional biomolecule involved in a variety of physiological and pathological processes. Multiple studies have suggested the implication of NO in neuronal cell death in the context of neurodegenerative diseases, including PD [18,19,20]. NO has been shown to mediate 1-methyl-4-phenylpyridinium or 6-hydroxydopamine-induced DA neurotoxicity [21,22]. In a rodent model of PD induced by exposure to paraquat/maneb, DA neuronal cell death results from the production of NO [23]. Abatement of nitrosative stress prevents DA neurodegeneration in a combination of neuroinflammation with α-synuclein (α-syn) mutation-induced cellular and transgenic mice models of PD [24,25]. Furthermore, NO can cause S-nitrosylation of parkin to compromise its neuroprotective function via impairing the transcriptional repression of p53 gene [23].

The interactions between genetic predisposition and environmental stressors are thought to contribute to the demise of nigral DA neurons in idiopathic PD [26]. It has been demonstrated that inflammation, as an underlying factor in the pathogenesis of PD, plays a causal role in the interplay between genetics and environment [24,25,27]. Frank-Cannon et al. [27] reported that parkin deficiency increases vulnerability to inflammation-related nigral degeneration in systemic LPS treated-parkin-deficient (parkin−/−) mice. However, the mechanisms underlying the neuroprotective capacity of parkin response to inflammatory mediators, such as NO, are not yet clearly understood. Furthermore, the susceptibility of pathogenic parkin mutants to nitrosative stress-induced neurodegeneration also remains to be established. Thus, in the present study, a NO exposure-induced SH-SY5Y cell death model was used to investigate the effect of parkin and its pathogenic mutants on cell survival as well as to delve into the molecular mechanisms underlying this effect.

## 2. Results

### 2.1. Pathogenic Mutation of Parkin Impairs the Protective Capacity against NO-Induced Apoptosis

**Figure 1:**
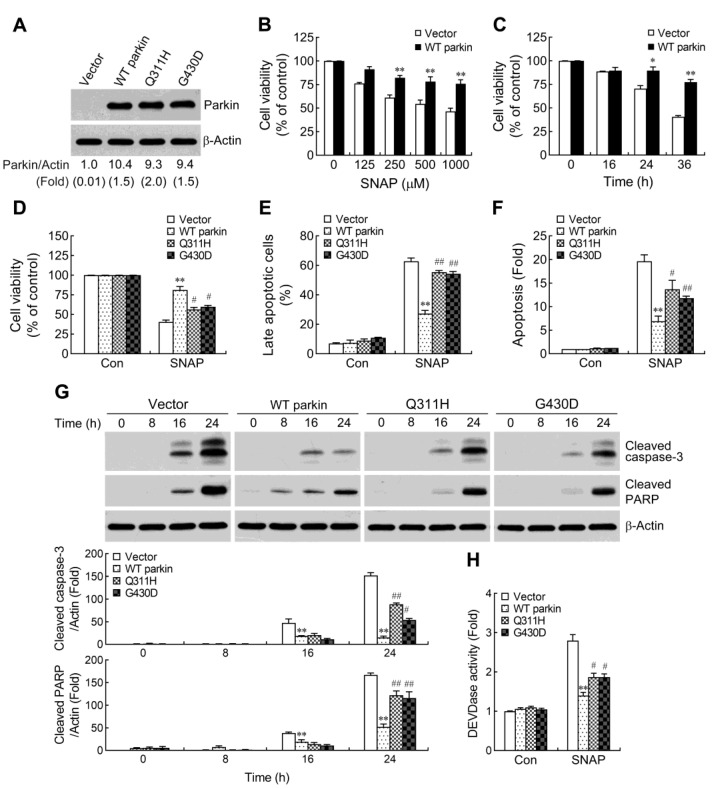
Wild-type (WT) parkin overexpression results in increased resistance to nitric oxide (NO)-induced apoptotic cell death. (**A**) Generation of SH-SY5Y clones overexpressing wild-type (WT) parkin and mutated parkin. SH-SY5Y cells were stably transfected with empty vector, WT parkin and the Q311H and G430D mutated parkin constructs. Whole-cell lysates were prepared from cells. Parkin expression was determined by immunoblotting. β-Actin was used as a loading control. Numbers below each lane show the fold increase relative to the vector control cells. Quantitative data are expressed as mean (top) ± SEM (brackets) of three separate determinations. (**B**,**C**) Control and WT parkin overexpressing cells were treated with various concentrations of the NO donor SNAP for 30 h (**B**) or treated with 1 mM SNAP for the indicated times (**C**). The cell viability was assessed by MTT. Data are presented as mean ± SEM for three independent experiments. * *p* < 0.05; ** *p* < 0.01 compared with respective vector control cells at each dose (**B**)/time point (**C**). (**D**–**F**) Pathogenic parkin mutations impair the protective potential of parkin. Cells were exposed to 1 mM SNAP for 30 h. Cell viability was determined using MTT assay (**D**). Cell apoptosis was analyzed by flow cytometry with Annexin V-FITC and propidium iodide (PI) double staining. Percentages of cells positive for both annexin V and PI are shown (**E**). (**F**) After treatment with SNAP, the nucleosomes in cell lysates were also quantified for apoptosis using a cell death detection ELISA kit. Values were expressed relative to the non-stimulated vector control cells. Data are presented as mean ± SEM for three independent experiments. ** *p* < 0.01 compared with vector control cells treated with SNAP. ^#^ *p* < 0.05; ^##^ *p* < 0.01 compared with WT parkin-expressing cells treated with SNAP. (**G**,**H**) Cells were treated with 1 mM SNAP for the indicated periods (**G**) or 24 h (**H**). Whole-cell lysates were prepared and subjected to Western blot analysis with antibodies against cleaved caspase-3 and cleaved poly (ADP-ribose) polymerase (PARP) (**G**). The blots from four cells were exposed to the same X-film as described in the Materials and Methods section. The graphs show the quantification analysis of cleaved caspase-3 and cleaved PARP/β-actin. Caspase-3-like protease activity (**H**) in cell lysates was measured by cleavage of the fluorogenic substrate Ac-DEVD-AMC. Values were expressed relative to the non-stimulated vector control cells. Data are presented as mean ± SEM for four independent experiments. ** *p* < 0.01 compared with respective vector control cells treated with SNAP at each time point. ^#^ *p* < 0.05; ^##^ *p* < 0.01 compared with respective WT parkin-expressing cells treated with SNAP at each time point. * or # indicates *p* value is less than 0.05; ** or ## indicates *p* value is less than 0.01.

Human neuroblastoma SH-SY5Y cells have been extensively used as an in vitro model to explore the cellular and molecular mechanisms underlying the pathogenesis of PD [28]. The endogenous level of parkin expression is very low in this cell line [23], allowing us to easily identify the function of wild-type (WT) or mutated parkin overexpression under stress conditions. Furthermore, SH-SY5Y cells have been used in studies of parkin function [9,10,23]. To assess the influence of parkin expression on NO-induced cell death, we generated stable clones of SH-SY5Y cells overexpressing WT and mutated parkin (Q311H and G430D) [9,29]. Immunoblot analyses revealed that parkin protein levels were comparable (no significant difference) in cells overexpressing WT or mutated parkin (Figure 1A). To evaluate the effect of WT parkin on cell viability under NO exposure, we treated control and WT parkin overexpressing cells with various concentrations of NO donor *S*-nitroso-*N*-acetyl-DL-penicillamine (SNAP) for 30 h, or at 1 mM for different periods. As shown in Figure 1B,C, SNAP induced a dose and time-dependent reduction of cell viability. Furthermore, overexpression of WT parkin rendered the cells more resistant to SNAP exposure by significantly decreasing SNAP-induced cell death. We next examined whether parkin mutations associated with AR-JP could impair the protective phenotype elicited by WT parkin. Indeed, the pathogenic parkin mutants Q311H and G430D showed a reduced protective capacity (Figure 1D). To further confirm whether WT parkin promotes and pathogenic parkin mutants impair the protective potential against NO-induced cell death, we used sodium nitroprusside (SNP) as another exogenous NO donor compound to determine its effect on cell death. Similarly, the defective protection by parkin mutants was observed following SNP treatment (Figure S1A). Moreover, the discrepancy between WT and mutant parkin overexpressing cells was not due to the different release of NO in the culture medium (Figure S1B). NO has been shown to induce apoptotic neuronal death [18,23]. To detect cell apoptosis, SH-SY5Y cells were analyzed by flow cytometry method after staining with Annexin V and PI (Figure 1E and Figure S1). The results revealed that SNAP-induced apoptosis was reduced in WT parkin overexpressing cells compared with that in vector control cells. We further confirmed these results with a cell apoptosis detection ELISA, which specifically detects the DNA fragmentation and histone release, two specific products of apoptosis [30]. Consistent with the observation of an increased proportion of apoptotic cells in vector control, SNAP treatment resulted in augmented release of apoptosis products in these cells compared to WT parkin overexpressing cells. (Figure 1F). Similarly, the parkin mutants were also impaired in protecting cells from SNAP-induced cell apoptosis (Figure 1E,F). 

Activation of caspases constitutes a crucial mechanism for induction of apoptosis. To determine whether SNAP-induced apoptosis is associated with caspases activation, we assessed at different time points the cleaved form of caspase-3, a critical executioner of apoptosis, by Western blotting. As shown in Figure 1G, SNAP-induced caspase-3 cleavage was increased with time in cells, and the level of cleaved-caspase-3 was higher in vector control cells compared with that in WT parkin overexpressing cells. Moreover, cleavage of poly (ADP-ribose) polymerase (PARP), one of the main cleavage targets of caspase-3, also serves as a marker of cells undergoing apoptosis. Again, WT parkin overexpression decreased the cleaved-PARP levels following SNAP exposure (Figure 1G). Measurement of caspase-3 activity with a specific fluorogenic substrate, DEVD-AMC, further confirmed that overexpression of WT parkin reduced the activation of caspase-3 by SNAP treatment (Figure 1H). However, parkin mutants were defective in attenuating SNAP-induced activation of caspase-3 cascade (Figure 1G,H).

### 2.2. ROS Reduction Contributes to Parkin-Mediated Abatement of Apoptosis by NO 

**Figure 2:**
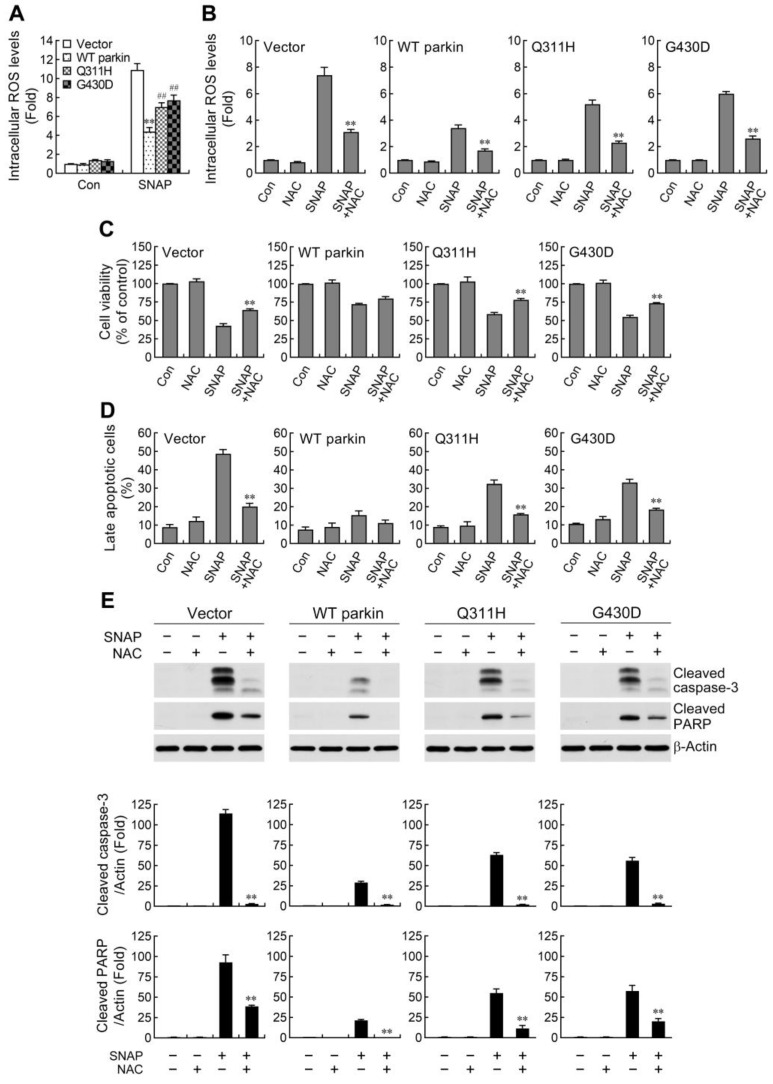
Reduced reactive oxygen species (ROS) production conduces to the protective effects of WT parkin overexpression in NO-induced apoptosis. (**A**,**B**) Cells were incubated with 1 mM SNAP for 24 h in the absence (**A**) or presence of *N*-acetyl-L-cysteine (NAC, 5 mM) (**B**). Intracellular ROS levels were measured using carboxy-H_2_DCFDA by performing flow cytometry, and the mean fluorescence intensity was used to represent relative ROS. Values were expressed relative to the non-stimulated vector control cells (**A**) or respective control cells (**B**). Data are presented as mean ± SEM for three independent experiments. In (**A**), ** *p* < 0.01 compared with vector control cells treated with SNAP. ^##^ *p* < 0.01 compared with WT parkin-expressing cells treated with SNAP. In (**B**), ** *p* < 0.01 compared with SNAP alone. (**C**,**D**) Abatement of ROS increases cell survival and mitigates apoptosis following NO exposure. Cells were pretreated with NAC (5 mM) for 1 h followed by exposure to SNAP for another 30 h. Cell viability and apoptosis were analyzed by MTT (**C**) and Annexin V-FITC staining (**D**), respectively, as described in Figure 1. Data are presented as mean ± SEM for four independent experiments. ** *p* < 0.01 compared with SNAP alone. (**E**) Blockade of ROS production reduces NO-induced caspase-3 cascade activation. Western blot of cleaved caspase-3 and cleaved PARP proteins in cell lysates from cells pretreated with 5 mM NAC followed by treatment with 1 mM SNAP for 24 h. Values are expressed relative to the respective control cells. Data are presented as mean ± SEM for three independent experiments. ** *p* < 0.01 compared with SNAP alone.

Previous studies have demonstrated that NO induced apoptosis by increasing the levels of ROS in various cell types [31,32]. Therefore, we evaluated the effect of WT and mutant parkin overexpression on SNAP-caused increase in intracellular ROS. Our results showed that the level of intracellular ROS was higher in vector control cells following SNAP exposure (Figure 2A). Overexpression of WT parkin was observed to block the elevation of ROS, while mutant parkin showed defective suppression of ROS production. To further confirm whether elevated ROS play a role in SNAP-induced cell death, cells were treated with *N*-acetyl-L-cysteine (NAC), an intracellular ROS scavenger, prior to SNAP exposure. The increased levels of SNAP-induced intracellular ROS were diminished following NAC treatment (Figure 2B). Moreover, cell death and apoptosis caused by SNAP were significantly prevented by NAC in both vector control cells and mutant parkin overexpressing cells but only slightly reduced in WT parkin overexpressing cells (Figure 2C,D and Figure S2). Based on the foregoing evidence that SNAP induced apoptosis through increasing the activation of caspase-3 pathway, we further examined the contribution of ROS in this apoptotic process in cells. Consistent with the observed protection afforded by NAC addition, caspase-3 cascade activation induced by SNAP was largely abated in vector control cells and parkin mutants (Figure 2E). Altogether, our data indicate that increased resistance of WT parkin overexpressing cells to NO-induced apoptosis is attributed to, at least partially, the reduction of ROS.

### 2.3. Parkin Selectively Upregulates IRE1α/XBP1 Signaling in Response to NO

**Figure 3:**
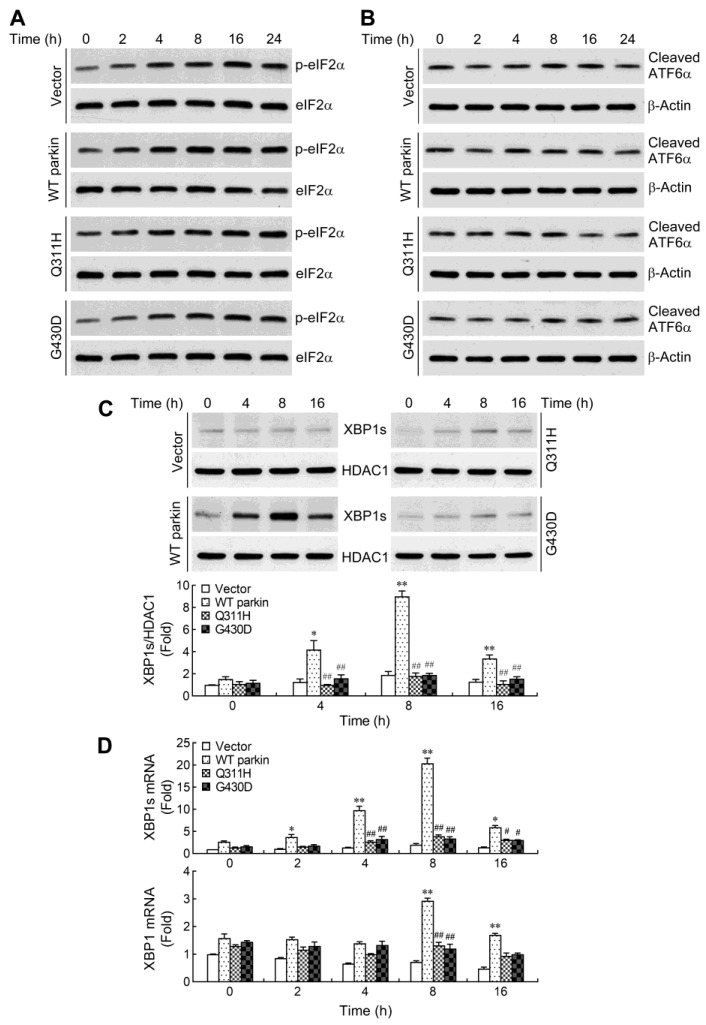
Wild-type parkin overexpression selectively activates inositol-requiring enzyme 1α/X-box binding protein 1 (IRE1α-XBP1) pathway in response to NO exposure. Cells were treated with 1 mM SNAP for the indicated times. Whole-cell (**A**,**B**) and nuclear (**C**) extracts were prepared and subjected to Western blotting using antibodies specific for phosphorylated eIF2α, ATF6α (recognized cleaved form) and spliced XBP1 (XBP1s). Total forms of eIF2α, β-actin and HDAC1 immunoblotting were performed to monitor loading. The blots from four cells were exposed to the same X-film. (**D**) Total/spliced XBP1 mRNA expression was determined by real-time RT-PCR. Values were expressed relative to the non-stimulated vector control cells. Data are presented as mean ± SEM for three independent experiments. * *p* < 0.05; ** *p* < 0.01 compared with respective vector control cells treated with SNAP at each time point. ^#^ *p* < 0.05; ^##^ *p* < 0.01 compared with respective WT parkin-expressing cells treated with SNAP at each time point.

Disturbance in endoplasmic reticulum (ER) homeostasis culminates in ER stress. In mammals, three unfolded protein response (UPR) pathways have evolved to recover or maintain ER function following ER stress. These pathways are initiated by three ER transmembrane sensors: protein kinase R (PKR)-like ER kinase (PERK), activating transcription factor 6 (ATF6), and inositol-requiring enzyme 1α (IRE1α) [33,34]. However, if the UPR fails and ER stress persists, specialized apoptotic pathways are activated and result in cell death. It has been shown that induction of C/EBP homologous protein (CHOP), an ER stress-associated apoptotic molecule, contributes to NO-induced apoptosis in a range of cell types [35,36,37]. Therefore, we further determined whether ER stress occurs and leads to apoptosis of SH-SY5Y cells under NO exposure. We first analyzed changes in the protein levels of three UPR signaling pathways after SNAP treatment in SH-SY5Y cells. Activation of the PERK/eIF2α axis was determined by measuring phosphorylation of eIF2α, a direct target of PERK. Following treatment with SNAP, the levels of phosphorylated eIF2α in four cells were increased with time, but the increase in phosphorylation of eIF2α was not significantly different among those treated cells (Figure 3A). In addition, the PERK/eIF2α-mediated expression of CHOP is the main pathway of ER stress-triggered apoptosis [38]. We next examined whether the protein levels of CHOP changed upon treatment with SNAP but found no induction (data not shown). Upon ATF6 branch UPR activation, ATF6α is cleaved to a smaller form (50 kDa) at the Golgi apparatus. We found no change in the cleavage of ATF6α throughout the time course of SNAP treatment in all cells (Figure 3B). Autophosphorylation of IRE1α results in splicing of the X-box binding protein 1 (XBP1) mRNA to produce a small fragment mRNA, which is translated to an active form of transcriptional factor [39]. Therefore, activation of IRE1α/XBP1 pathway was manifested as accumulation of spliced XBP1 (XBP1s) protein in the nucleus. After exposure to SNAP, the overexpression of WT parkin resulted in a larger increase in XBP1s protein levels compared to vector control cells (Figure 3C). In line with the increased production of XBP1s protein, enhanced expression of XBP1s mRNA was also observed in WT parkin overexpressing cells (Figure 3D). A previous demonstration that parkin enhanced XBP1 gene transcription [40] led us to determine whether WT parkin-mediated augmentation of XBP1 mRNA expression also occurs in NO-mediated stress condition. The results showed that an upregulated XBP1 mRNA expression was detected until later time-points (8 h) (Figure 3D). In contrast, there was no obvious increase of XBP1s protein and mRNA in parkin mutants. Taken together, our results suggest that CHOP is not a mediator in NO-induced cell death and overexpression of WT parkin selectively upregulates IRE1α/XBP1 signaling.

### 2.4. IRE1α/XBP1 Signaling Conduces to Parkin-Mediated Cell Protection 

**Figure 4:**
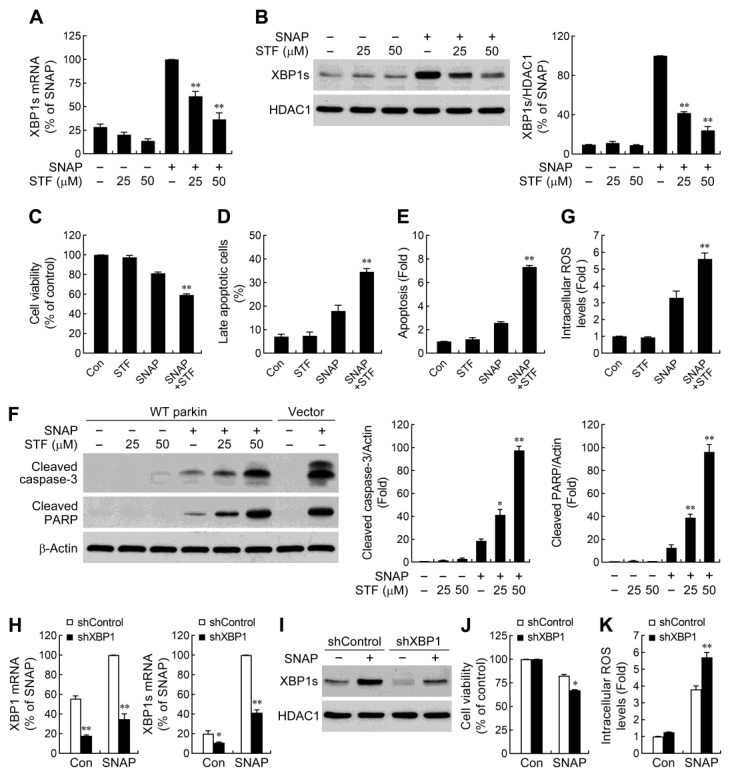
Increased XBP1 splicing contributes to WT parkin overexpression-enhanced resistance to NO-induced cell apoptosis. (**A**,**B**) WT parkin overexpressing cells were pretreated with 25 and 50 μM STF-083010, a selective IRE1α RNase inhibitor, for 1 h followed by exposure to 1 mM SNAP for another 8 h. The expression of XBP1s mRNA (**A**) was quantified by real-time RT-PCR. Spliced XBP1 protein levels (**B**) were assessed in the nuclear fractions by Western blot analysis. Data are presented as mean ± SEM for four independent experiments. ** *p* < 0.01 compared with SNAP alone. (**C**–**E**) Inhibition of XBP1 splicing in WT parkin overexpressing cells aggravates NO-induced cytotoxicity. WT parkin overexpressing cells were pretreated with 50 μM STF-083010 for 1 h followed by exposure to SNAP for another 30 h. Cell viability and apoptosis caused by SNAP were assessed, as described in Figure 1. Data are presented as mean ± SEM for four independent experiments. ** *p* < 0.01 compared with SNAP alone. (**F**) WT parkin overexpressing cells were treated with SNAP for 24 h in the absence or presence of STF-083010 (25 and 50 μM). Whole-cell extracts were subjected to immunoblot analysis with antibodies against cleaved caspase-3 and cleaved PARP. Data are presented as mean ± SEM for four independent experiments. * *p* < 0.05; ** *p* < 0.01 compared with SNAP alone. (**G**) Attenuation of XBP1 splicing impairs WT parkin-mediated suppression of NO-induced ROS production. Twenty-four hours after treatment with SNAP plus STF-083010 (50 μM), WT parkin overexpressing cells were stained with carboxy-H_2_DCFDA and then subjected to flow cytometry for determination of intracellular ROS. Data are presented as mean ± SEM for three independent experiments. ** *p* < 0.01 compared with SNAP alone. (**H**–**K**) XBP1 knockdown sensitizes cells to NO-induced cell death. WT parkin overexpressing cells were transduced with lentivirus carrying shRNA targeting XBP1 (shXBP1) or the scramble control sequence (shcontrol). Stable clones of each group were selected. Both clones were treated with 1 mM SNAP for 8 h (**H**,**I**), 30 h (**J**), or 24 h (**K**). Total RNA and nuclear proteins were extracted. Expression levels of XBP1/XBP1s mRNA (**H**) and protein (**I**) were analyzed by real-time RT-PCR and Western blotting, respectively. Cell viability (**J**) was assessed by MTT. The levels of intracellular ROS (**K**) were determined as described in (**G**). Data are presented as mean ± SEM for four independent experiments. * *p* < 0.05; ** *p* < 0.01 compared with respective control shRNA-expressing cells untreated or treated with SNAP.

IRE1α/XBP1 pathway is an important mechanism, which protects various cell types from apoptosis [41,42,43,44,45]. Upon activation, IRE1α splices XBP1 mRNA through an unconventional mechanism using its endoribonuclease (RNase) activity [39]. To determine whether parkin-regulated cell survival is associated with the IRE1α/XBP1 pathway, we used an IRE1α RNase inhibitor STF-083010 to compromise the production of spliced XBP1 in SNAP-treated WT parkin overexpressing cells. STF-083010 is a specific inhibitor which binds to the RNase domain of IRE1α and inhibits the cleavage of XBP1 mRNA, while the kinase activity is left intact [46,47]. We found that inhibition of IRE1α RNase activity by STF-083010 limited SNAP-induced XBP1 mRNA splicing (Figure 4A) and the nuclear accumulation of XBP1s protein (Figure 4B). We next examined the potential role of XBP1s in WT parkin overexpression-induced effects on cell survival following SNAP exposure. The results showed that reduction of XBP1s levels by STF-083010 decreased cell viability (Figure 4C) and exacerbated cell apoptosis (Figure 4D,E and Figure S2). Consistent with the observed deleterious effects afforded by XBP1s loss, the caspase-3 cascade activation induced by SNAP was also found to be increased in STF-083010-treated cells (Figure 4F). XBP1 has been shown to be associated with oxidative stress [44,48]. To investigate whether reduced ROS production in WT parkin overexpressing cells is due to the increase of XBP1s, we measured the SNAP-induced ROS generation following blockade of the XBP1 splicing by STF-083010. Indeed, STF-083010 treatment markedly augmented SNAP-induced ROS production (Figure 4G), indicating that enhanced XBP1 splicing was involved in parkin-mediated reduction of ROS levels.

The findings that XBP1s was responsible for parkin overexpression-enhanced cell viability elucidated from a pharmacological approach were further confirmed by genetic manipulation experiments. We downregulated XBP1s levels (Figure 4H,I) by introducing XBP1 targeted shRNAs into WT parkin overexpressing cells and then assessed their effects on cell survival. As shown in Figure 4J, the protective effect of WT parkin upon SNAP-induced cell death was dampened by XBP1 shRNA. Moreover, XBP1 knockdown increased the intracellular ROS levels (Figure 4K). These findings collectively suggest that parkin enhances cell survival under NO exposure through IRE1α/XBP1 signaling-mediated ROS reduction.

### 2.5. Parkin Enhances IRE1α Phosphorylation and Oligomerization

**Figure 5:**
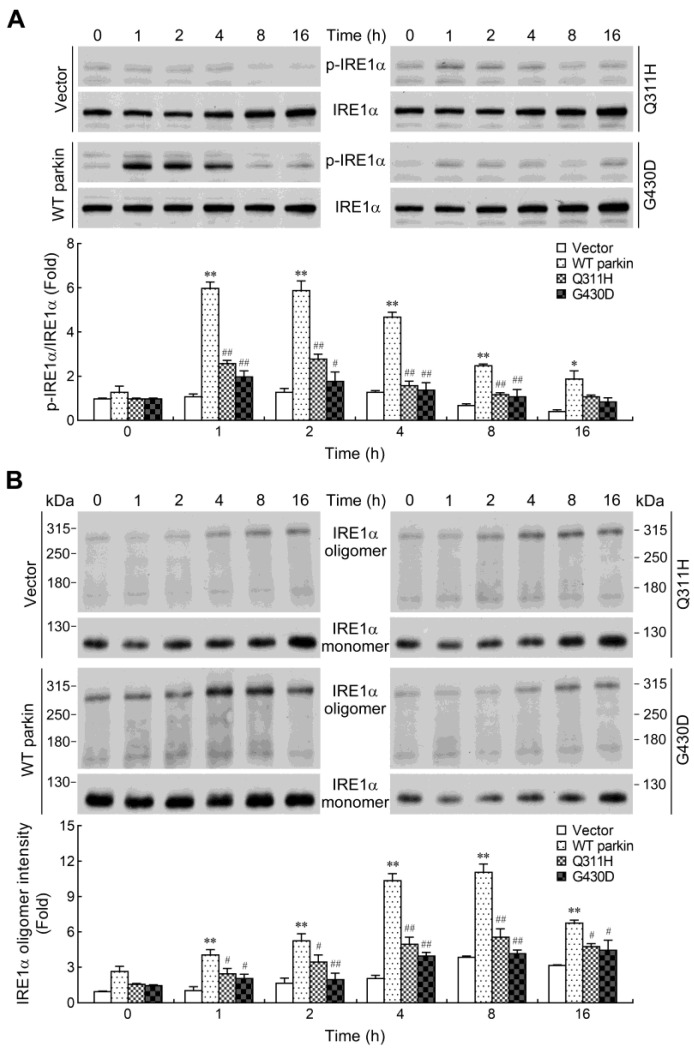

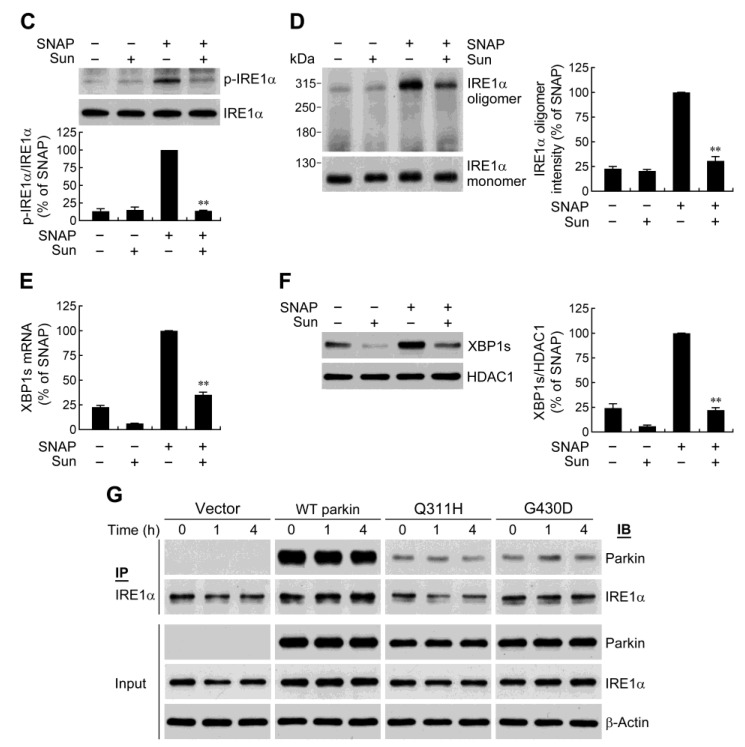
Parkin augments IRE1α phosphorylation/oligomerization-mediated XBP1 splicing. (**A**,**B**) Cells were exposed to SNAP (1 mM) for the indicated times. Western blot analysis was used to determine phosphorylated IRE1α (Ser724) protein in whole-cell lysates (**A**). Otherwise, the cell lysates were separated by SDS-PAGE in the absence of the reducing agent DTT (**B**). Immunoblotting was performed using anti-IRE1α antibody. The band intensity of IRE1α oligomers was quantified with a densitometric analysis. The blots from four cells were exposed to the same X-film. Values were expressed relative to the non-stimulated vector control cells. Data are presented as mean ± SEM for four independent experiments. * *p* < 0.05; ** *p* < 0.01 compared with respective vector control cells treated with SNAP at each time point. ^#^ *p* < 0.05; ^##^ *p* < 0.01 compared with respective WT parkin-expressing cells treated with SNAP at each time point. (**C**–**F**) Suppression of IRE1α phosphorylation/oligomerization reduces XBP1 splicing. WT parkin overexpressing cells were pretreated with 5 μM sunitinib (Sun) for 1 h prior to SNAP treatment for 1 h (**C**), 4 h (**D**) or 8 h (**E**,**F**). Western blot analysis of p-IRE1α (**C**), IRE1α oligomers (**D**) and XBP1s proteins (**F**) were carried out as above. The expression of XBP1s mRNA € was quantified by real-time RT-PCR. Data are presented as mean ± SEM for four independent experiments. ** *p* < 0.01 compared with SNAP alone. (**G**) Parkin complexes with IRE1α. Cells were treated with 1 mM SNAP for 1 h and 4 h. Cell lysates were immunoprecipitated (IP) with anti-IRE1α antibody and then subjected to immunoblotting (IB) analyses with anti-parkin antibody. The cell extracts were also blotted for IRE1α, parkin and β-actin. The blots from four cells were exposed to the same X-film. The immunoblots are representative of four independent experiments.

As mentioned above, the differences in XBP1 splicing were apparent in WT parkin overexpressing cells as early as 2 h post SNAP treatment while increased XBP1 mRNA levels was not observed until later time-points (8 h) (Figure 3D). The results suggested that an additional regulation might be involved in WT parkin-enhanced XBP1s mRNA expression. Therefore, we envisioned the possibility that parkin might augment the RNase activity of IRE1α following SNAP treatment. IRE1α consists of a luminal N-terminal domain inside the ER lumen and two functional cytoplasmic domains having kinase and RNase activities. The activation of IRE1α requires dimerization, which then triggers its autophosphorylation and leads to the activation of its RNase domain [49,50,51]. Phosphorylation of kinase domain residues Ser724 and Ser726 has been shown to make the greatest contribution to the increase in RNase activity [51]. To test the effect of WT parkin on SNAP-induced IRE1α activation, immunoblot assays were performed to determine the phosphorylation state of this protein. The results showed that a higher level of phosphorylated IRE1α (S724) was found as early as 1 h and resolved by 8 h in WT parkin overexpressing cells, whereas the parkin mutants had decreased potential to induce IRE1α phosphorylation (Figure 5A).

Besides the key step of dimerization/autophosphorylation in IRE1α activation, oligomerization of IRE1α is also shown to correlate with RNase activation and XBP1 splicing [52,53]. To determine whether IRE1α oligomerization is enhanced by overexpressing WT parkin, we used a nonreducing SDS-PAGE to examine the oligomerization state in SNAP-treated cells. As shown in Figure 5B, in the absence of the reducing agent DTT, both monomeric and oligomeric forms of IRE1α was observed. Based on the demonstration that IRE1α oligomerization can improve XBP1 splicing, the band intensity of IRE1α oligomers was quantified with a densitometric analysis. Exposure of cells to SNAP resulted in an increase in IRE1α oligomers. Consistent with the divergent induction of IRE1α phosphorylation in those cells, WT parkin overexpression also significantly elevated the levels of IRE1α oligomers.

To examine the participation of phosphorylation/oligomerization on the IRE1α RNase activity in WT parkin overexpressing cells following SNAP exposure, we analyzed the induction of XBP1s in the presence of an ATP-competitive inhibitor sunitinib. Sunitinib has been shown to inhibit phosphorylation of IRE1α and block consequent RNase activity [49]. We found that sunitinib was able to suppress the increase in phosphorylation and oligomerization of IRE1α (Figure 5C,D). At the same time, the expressions of XBP1s mRNA and protein were also shown to be markedly attenuated by this inhibitor (Figure 5E,F). These results indicate that IRE1α phosphorylation/oligomerization is essential for parkin-mediated XBP1 mRNA splicing in response to NO, and this activity is collapsed with mutant parkin.

### 2.6. Parkin Forms a Protein Complex with IRE1α

There is now accumulating evidence to suggest that the IRE1α activity is controlled by a complex protein platform, known as the UPRosome, at the ER membrane [54,55]. A few proteins have been identified to regulate IRE1α dimerization/oligomerization through protein-protein interactions [56,57,58]. Thus, we explored the possibility of an interaction between parkin and IRE1α. Cells were treated with SNAP, and the complexes of parkin-IRE1α in cell lysates were determined by co-immunoprecipitation assays. The parkin-IRE1α complex was detected in WT and mutant parkin overexpressing cells under control conditions (Figure 5G). While an interaction was not observed in vector control cells, that was possibly due to the rare expression of parkin in this cell line [23]. Further, the association of parkin with IRE1α was not affected in cells undergoing SNAP stimulation. Interestingly, we found that mutant parkin impaired its binding to IRE1α. To further clarify whether the interaction of IRE1α with parkin is due to a nonspecific binding resulting from overexpressed parkin, we carried out the IP experiments using PC12 cells, which also widely used to study PD and physiologically express parkin proteins. We were also successful in detecting a physical association between endogenous parkin and endogenous IRE1α (Figure S3). These findings suggest that augmentation of IRE1α activity by parkin might be mediated through interacting with IRE1α to regulate its phosphorylation/oligomerization processes.

## 3. Discussion

Growing evidence suggests that PD may be caused by interactions among multiple factors, including genetic predisposition, environmental toxins exposure and neuroinflammation. Several environmental toxins, such as MPTP, rotenone, and paraquat, are known to promote neuroinflammatory responses [59,60,61]. Consequently, insights into the interplay of PD-associated genes and neuroinflammation may facilitate a better understanding of the pathogenesis of PD. The inflammation-mediated neurotoxic effects are attributed to the secretion of proinflammatory and cytotoxic factors induced by activated microglia. Several lines of evidence indicate the involvement of NO in the apoptotic cell death of DA neurons that occurs in PD [18,19,21]. Since mutations in the parkin gene account for most AR-JP [3,4], we herein used a NO exposure-induced cell death model to elucidate the contribution of pathological parkin combined with nitrosative stress in cell death. Our findings showed that overexpression of WT parkin in SH-SY5Y cells reduced cell death after SNAP (a NO donor) treatment, whereas the pathogenic parkin mutants Q311H and G430D appeared to have an impaired protective ability. These observations are in line with many publications showing that parkin exerts neuroprotective capacity against a remarkably wide array of neurotoxic stressors [6,7,8,9,10]. Previous studies reported that direct nigral or intraperitoneal administration of LPS results in a dramatic loss of DA neurons in human A53T mutant α-syn transgenic mice compared to WT mice [24,25]. Furthermore, knockout of parkin in mice renders nigral DA neurons more vulnerable to LPS-induced inflammation [27]. These demonstrations indicate that inflammation, acting as an environmental stressor, and genetic susceptibility synergistically promote progressive degeneration of DA neurons. Herein, we demonstrated the crucial role of genetic mutations-inflammatory insult interaction in induction of SH-SY5Y cell death that might be relevant to DA neurons. 

Even though NO is an essential molecule in neuronal signal transduction, excess NO can be neurotoxic. NO and reactive nitrogen species can cause ROS generation by inhibiting the activity of mitochondrial complexes [62,63,64]. In the current study, we found that the induction of ROS was increased in response to SNAP. Moreover, overexpression of WT parkin attenuated SNAP-induced ROS production, whereas parkin mutants exhibited an impaired effect on the decrease of ROS. Blockade of ROS elevation by NAC significantly suppressed SNAP-induced cell apoptosis in vector control cells and parkin mutants, while it had little significant effect on WT parkin overexpressing cells. This is consistent with other studies which found that ROS contributes to NO-induced cell apoptosis [31,32]. Our findings suggest that the differences in susceptibility to NO in these SH-SY5Y cells seem to be related, at least in part, to their endogenous levels of ROS. Additionally, the reduction of ROS levels might provide a part of the protective effect of WT parkin following NO exposure. Previous studies have demonstrated that WT parkin expression effectively abates the rise in ROS levels, while mutant parkin has a partial prevention effect on ROS production with or without neurotoxin exposure, and the discrepancy in ROS-lowering capacity is shown to be associated with their protective outcomes [65,66,67,68]. Our experiments further supported the proposal that parkin can modulate oxidative stress under various insults.

Although induction of CHOP has been implicated in NO-caused cell death [35,36,37], CHOP expression was not seen in our SNAP treatment systems. Unexpectedly, WT parkin overexpression could selectively activate the IRE1α/XBP1 pathway, leading to accumulation of XBP1s. The IRE1α/XBP1 pathway is one of the UPR signaling networks commonly activated by ER stress or by other physiological stimuli not limiting to lipid perturbation, immune response or viral infection [55,69]. XBP1 has been reported to play a role in protecting various cell types, including neurons [41,43,44,45,70,71,72]. In two different Alzheimer’s disease models, differentiated rat pheochromocytoma (PC12) cells treated with amyloid-β (Aβ) oligomers and flies expressing Aβ, XBP1s shows neuroprotective activity [43]. Furthermore, the activation of IRE1α/XBP1 signaling protects DA neurons against several toxin-associated PD-related insults in vitro and in vivo [45,70]. Overexpression of XBP1 has been shown to ameliorate DA neuron degeneration induced by α-syn in *Caenorhabditis elegans*, whereas neuron-specific knockdown of XBP1 aggravates the neurodegeneration process [73]. Therefore, XBP1 may act as a molecular target for modulating neuronal cell survival in neurodegenerative diseases. Indeed, when the levels of XBP1s were reduced by pharmacologically inhibiting IRE1α RNase activity or by genetically knocking down XBP1, WT parkin had reduced protective activity against NO-induced toxicity. Furthermore, pathogenic parkin mutants with abated protective ability were also compromised in their capacity to efficiently activate IRE1α/XBP1 signaling. It has been shown that parkin mediates neuroprotection via activation of IκB kinase/nuclear factor-κB signaling, transcriptional repression of p53 and induction of mitophagy [9,10,23,74]. Our findings indicate that activation of the IRE1α/XBP1 signaling axis is, at least partially, responsible for parkin overexpression-mediated prevention of SH-SY5Y cell death in response to NO exposure, and mutations of parkin disrupt this function. 

An association between XBP1 and oxidative stress has been demonstrated in a variety of cell types. Liu et al. [41] reported that XBP1 depletion renders mouse embryonic fibroblasts more sensitive to oxidative damage induced by H_2_O_2_ and induces a more extensive ROS generation. Ablation of XBP1 results in increased ROS levels and cell apoptosis in retinal pigment epithelium [44]; conversely, overexpression of XBP1 alleviates cigarette smoke extract-induced cell death [75]. In renal mesangial cells, high glucose-induced ROS production was suppressed by overexpression of XBP1s [48]. Furthermore, targeting XBP1 enhances sensitivity of glioma cells to oxidative stress [71]. Altogether, these findings suggest a role of XBP1 in regulation of oxidative stress in cells. In the present study, we found that pharmacological inhibition of XBP1 splicing or knockdown of XBP1 drastically increased SNAP exposure-induced ROS production in parkin overexpressing cells. Our results suggest that under NO exposure, parkin mediates its anti-oxidative function through, in part, the activation of the IRE1α/XBP1 pathway.

Here, we demonstrate that IRE1α/XBP1 signaling is specifically enhanced by WT parkin overexpression in response to an NO challenge. Autophosphorylation of IRE1α has been shown to correlate tightly with its RNase activity leading to augmented XBP1 splicing [49,50,51]. In agreement with this notion, WT parkin-enhanced IRE1α phosphorylation was observed following SNAP exposure. Inhibition of IRE1α phosphorylation by an ATP-competitive inhibitor sunitinib resulted in decrease of XBP1s levels, suggesting that increased phosphorylation of IRE1α is necessary for WT parkin-mediated augmentation of XBP1 splicing. Although IRE1α dimerization/autophosphorylation may possess RNase activity to splice XBP1 mRNA, IRE1 α oligomerization is thought to have maximal splicing efficiency [52,53]. Our findings revealed that overexpression of WT parkin elicited a more pronounced accumulation of IRE1α oligomers following SNAP treatment and the temporal accumulation profiles of IRE1α oligomers were parallel with the expression of XBP1s mRNA. Moreover, addition of sunitinib also reduced the formation of IRE1α oligomers, which might further contribute to the abrogation of XBP1 splicing. These findings indicate that parkin-enhanced formation of IRE1α oligomers might improve XBP1 splicing. Accordingly, the defective effect of mutant parkin on the activation of IRE1α/XBP1 signaling might be due to the lesser extent of IRE1α phosphorylation and oligomerization. Furthermore, our results also showed that overexpressed WT parkin triggered an increase of XBP1 mRNA expression, which might in turn expand the mRNA pool for splicing by IRE1α. It has been found that parkin upregulates the transcription of XBP1 gene in a p53-dependent manner under ER stress [40]. The potential contribution of p53 in the parkin-mediated increase of total XBP1 mRNA levels in NO-mediated stress condition merits further exploration.

Many proteins have been identified to modulate IRE1α signaling through protein-protein interactions [54,55]. Nevertheless, only a few proteins are known to bind IRE1α and positively regulate the IRE1α activity via modulating dimerization/oligomerization process. For example, ER stress-induced formation of ASK1-interacting protein 1-IRE1α complex has been shown to facilitate IRE1α dimerization/autophosphorylation [56]. The nonmuscle myosin IIB protein interacts with IRE1α, promoting the formation of larger IRE1α clusters [57]. Additionally, under ER stress, the Abelson tyrosine protein kinase 1 (ABL1 or c-abl) co-localizes with IRE1α at the ER membrane, thus driving high-order oligomerization, leading to hyperactivation of the RNase function [58]. Herein, we found that parkin also bound to IRE1α and the interaction was NO exposure-independent. A similar phenomenon has been reported: Hsp72 positively regulates IRE1α activity by forming a protein complex with IRE1α, and this interaction is not altered when undergoing ER stress [42]. Furthermore, overexpression of WT parkin resulted in a stronger association with IRE1α and increased IRE1α oligomers formation, whereas mutant parkin had impaired binding with IRE1α accompanied by a decrease of IRE1α oligomerization. Whether defective interaction with IRE1α in the parkin mutation is mediated by impairing E3 ubiquitin ligase activity or altering a binding motif is worth further investigation. Our findings suggest that parkin might act as a positive regulator of IRE1α activation by regulating its phosphorylation/oligomerization, which are possibly mediated through interacting with IRE1α. Nevertheless, the molecular mechanism of how WT parkin increases IRE1α phosphorylation/oligomerization-mediated XBP1 splicing, and whether the interaction of parkin with IRE1α is necessary for this function also require further exploration.

## 4. Materials and Methods

### 4.1. Materials

Cell culture ingredients were purchased from Invitrogen (Carlsbad, CA, USA). *S*-nitroso-*N*-acetyl-DL-penicillamine, sodium nitroprusside, STF-083010 and sunitinib were obtained from Sigma-Aldrich (St. Louis, MO, USA). Mouse anti-ATF6α was bought from Santa Cruz Biotechnology (Santa Cruz, CA, USA). Rabbit anti-phospho-IRE1α was obtained from Novus Biologicals (Centennial, CO, USA). All other antibodies were from Cell Signaling Technology (Beverly, MA, USA). All other reagents were purchased from Sigma-Aldrich.

### 4.2. Generation of Stable Cell Lines

Human neuroblastoma SH-SY5Y cells obtained from American Type Culture Collection (ATCC, Manassas, VA, USA) were used in this study. Wild-type human parkin cDNA (*PARK2*) (NCBI, Accession Number: AB009973) was subcloned into pCMV-Tag2B (Stratagene, La Jolla, CA, USA). Site-directed mutagenesis was performed to produce the G430D [9] and Q311H [29] parkin mutants using the QuikChange II Site-Directed Mutagenesis kit (Stratagene). Nucleotide sequencing was performed of all constructs. A control cell line was generated by transfecting pCMV-Tag2B into SH-SY5Y cells. For generating stable wild-type and mutant parkin expressing cell lines, 6-well plates of sub-confluent SH-SY5Y cells were transfected with cDNA constructs with Lipofectamine^TM^ 3000 Reagent (Invitrogen) according to the manufacturer’s instructions. After 1 day transfection, cells were selected for positive clones with limited dilution in the presence of 500 μg/mL of Geneticin (G418, Invitrogen), which completely killed untransfected cells. Individual clones were confirmed by Western blot analysis with anti-parkin antibody.

### 4.3. Cell Cultures and Drug Treatment

All SH-SY5Y cell lines were grown in minimum essential medium (MEM) with Earle’s salts and Ham’s F-12 nutrient mixture (1:1) supplemented with 10% fetal bovine serum (FBS), 2 mM L-glutamine, 1% non-essential amine acids, 100 U/mL penicillin, 100 μg/mL streptomycin, and 500 μg/mL G418 and incubated in a humidified atmosphere of 5% CO_2_ at 37 °C. Rat pheochromocytoma PC12 cell line was obtained from ATCC and cultured in Dulbecco’s modified Eagle’s medium with 10% FBS, 100 U/mL penicillin and 100 μg/mL streptomycin. Cultures were treated with SNAP, a NO donor, until they were harvested for various assays.

### 4.4. Lentiviral Transduction

For knockdown of XBP1, we generated stable subclones of wild-type parkin overexpressing cells with reduced levels of XBP1 by targeting XBP1 mRNA with short hairpin interfering RNA (shRNA) using lentiviral transduction. The lentiviral vectors, expressing shRNA against human XBP1 or scramble control sequence were purchased from National Core Facility for Manipulation of Gene Function/Genomic research Center, Academia Sinica (Taipei, Taiwan). The targeting sequence identified for human XBP1 is 5′-AGATCGAAAGAAGGCTCGAAT-3′. For lentiviral transduction, wild-type parkin overexpressing cells were incubated with the lentiviral particles (multiplicity of infection, 5) containing 4 μg/mL polybrene (hexadimethrine bromide) for 24 h, followed by medium change. All infected cells were selected with both G418 (500 μg/mL) and puromycin (1 μg/mL) until the stable knockdown of the XBP1 gene.

### 4.5. Cell Viability Assay

Cell viability was evaluated by modified 3-(4,5-dimethylthiozol-2-yl)-2,5-diphenyl-tetrazolium bromide (MTT) rapid colorimetric assay. MTT is converted in living cells into insoluble formazan, and the amount of formazan produced is proportional to the number of viable cells. Briefly, cells were cultured in 24-well plates and treated with various concentrations of SNAP for the indicated times. The cultures were then incubated for 1 h at 37 °C following addition of the MTT (0.3 mg/mL in DMEM). The blue formazan produced was solubilized with DMSO. The absorbance was measured at 550 nm using a microplate ELISA reader (Molecular Devices, Silicon Valley, CA, USA).

### 4.6. Apoptosis Assay

For apoptotic cell determination, cells were stained with Annexin V and propidium iodide (PI) using FITC Annexin V/Dead Cell Apoptosis kit (Invitrogen) according to the manufacturer’s protocol. After treatment, cells were harvested and washed once with cold PBS. Cells were then stained in a 100 μL of binding buffer containing Annexin V-FITC and PI for 15 min in the dark at room temperature. The reaction was stopped by adding 400 μL of binding buffer, and cells were immediately analyzed using the FACSCalibur flow cytometer (BD Bioscience, San Diego, CA, USA). 

Apoptosis was also assessed by detecting cytoplasmic histone-associated-DNA-fragments (mono- and oligonucleosomes) of the apoptotic cells [30] using the commercially available Cell Death Detection ELISA^PLUS^ kit (Roche, Mannheim, Germany) according to the manufacturer’s instructions. Briefly, 20 μL of cell lysates was transferred to a streptavidin-coated microplate and incubated with anti-histone antibody (biotin-labeled) and anti-DNA antibody (horse radish peroxidase-conjugated) for 2 h at room temperature. The color was then visualized by 2,2′-azino-bis(3-ethylbenzothiazoline-6-sulfonic acid), and absorbance value was measured at 405 nm. For each experiment, the amount of protein in the cell lysates was assessed in separate wells, and the absorbance of each sample was normalized to the protein content of the extracts. The results were expressed relative to the untreated vector control.

### 4.7. Measurement of Intracellular ROS

Intracellular ROS generation was assessed using carboxy-H_2_DCFDA (Molecular Probes, Eugene, OR, USA). After treatment with SNAP for 24 h, cells were loaded with 10 μM carboxy-H_2_DCFDA in the dark at 37 °C for 30 min. The cells were then washed with PBS, harvested by trypsinization, and analyzed with flow cytometry with the excitation source at 488 nm and emission at 525 nm.

### 4.8. Real-Time RT-PCR Analysis

Total RNA was extracted from SH-SY5Y cells with TRIzol^®^ reagent (Invitrogen). After synthesis of cDNA from total RNA using SuperScript^TM^ III Reverse transcriptase (Invitrogen), SYBR Green chemistry in conjunction with real-time PCR was used to determine the expression of genes (*Power* SYBR^®^ Green PCR Master Mix, Applied Biosystems, Foster City, CA, USA). The primer sequences are as follows: XBP1, 5′-TGAGCTGGAACA GCAAGTGGT-3′ and 5′-CCCAAGCGCTGTCTTAACTCC-3′; spliced XBP1 (XBP1s), 5′-CTGAGTCCGCAGCAGGTG -3′ and 5′-AGTTGTCCAGAATGCCCAACA -3′; β-actin, 5′-CTGGGTATGGAATCTTGC-3′ and 5′-GTTGGCGTACAGGTCTTT-3′. Threshold cycle (C_t_) value for each test gene was normalized to the C_t_ value for the β-actin control from the same RNA preparations. The ratio of transcription of each gene was calculated as 2^−(ΔCt)^, where ΔC_t_ is the difference C_t(test gene)_ − C_t(β-actin)_.

### 4.9. Western Blotting

Whole-cell lysates or nuclear extracts were prepared from four cells by using the M-PER^®^ mammalian protein extraction reagent or the NE-PER^®^ nuclear and cytoplasmic extraction reagents (both from Thermo Scientific, Waltham, MA, USA), respectively, as per the manufacturer’s instructions. The protein concentration of samples was determined by Bradford assay (Bio-Rad, Hercules, CA, USA). Each set of protein samples was simultaneously subjected to immunoblotting. Equal amounts of protein were separated on 6~12% sodium dodecyl sulphate-polyacrylamide gel (SDS-PAGE) and transferred to immobilon polyvinylidene difluoride (PVDF) membranes (Merck Millipore, Billerica, MA, USA). The membranes were incubated in Tris-buffered saline Tween 20 (TBST, 0.1 M Tris/HCl, pH 7.4, 0.9% NaCl, 0.1% Tween 20) supplemented with 5% dry skim milk for 1 h to block nonspecific binding. After rinsing with TBST buffer, the membranes were incubated with the following antibodies: rabbit anti-parkin, rabbit anti-cleaved caspase-3, rabbit anti-cleaved PARP, rabbit anti-phospho-eIF2α (Ser51), rabbit anti-eIF2α, mouse anti-ATF6α, rabbit anti-XBP1s, rabbit anti-phospho-IRE1α (Ser724), rabbit anti-IRE1α, mouse anti-HDAC1, and mouse anti-β-actin. The membranes were washed three times with TBST buffer followed by incubation with appropriate horseradish peroxidase-conjugated secondary antibodies. The antigen-antibody complex was detected by using an ECL chemiluminescence detection system (PerkinElmer, Boston, MA, USA). The blots of four cells from each experiment were exposed to the same X-film. The intensity of the bands was quantified with a GS-900^TM^ calibrated densitometer (Bio-Rad) and calculated as the optical density X area of bands.

### 4.10. Immunoprecipitation

Whole-cell lysates (160 μg of protein) were incubated with a rabbit anti-IRE1α antibody (Santa Cruz Biotechnology) with gentle rocking overnight at 4 °C. PureProteome^TM^ protein G magnetic beads (Merck Millipore) were added (15 μL of suspension) and rotated for 3 h at 4 °C. After washing the beads with ice-cold immunoprecipitation buffer (20 mM Tris, pH 7.5, 150 mM NaCl, 1 mM EDTA, 1 mM EGTA, 1% Triton X-100, 2.5 mM sodium pyrophosphate, 1 mM β-glycerolphosphate, 1 mM Na_3_VO_4_, 1 μg/mL leupeptin, 1 mM PMSF), immunoprecipitated proteins were eluted in boiling SDS sample buffer, and subjected to Western blotting analyses.

### 4.11. Caspase Activity Assay

The caspase-3-like activity was determined using the Caspase-3 Activity Assay kit (Cell Signaling Technology) according to the manufacturer’s protocol. Briefly, 50 μL of lysates were added to 150 μL of assay buffer containing a fluorogenic substrate [1 mg/mL *N*-acetyl-Asp-Glu-Val-Asp-(7-amino-4-methylcoumarin)(Ac-DEVD-AMC)], which was cleaved by caspase-3. Accumulation of AMC fluorescence was monitored over 180 min using an FLx800 fluorescent plate reader (BioTek, Winooski, VT, USA) at excitation wavelength 380 nm and emission wavelength 460 nm. Fluorescence of each sample reading at time 0 h was subtracted from the detected value. AMC fluorescence (relative fluorescence units, RFU) of each sample was normalized to the protein content of the extracts. Caspase-3-like activity from the untreated sample was arbitrarily set at 1 for the calculation of fold induction.

### 4.12. NO Assay

NO release was assayed by measuring the concentrations of the stable NO metabolite, nitrite, in the conditioned medium. Briefly, 100 μL of culture supernatant were reacted with an equal volume of Griess reagent (1 part 0.1% naphthylethylenediamine, 1 part 1% sulfanilamide in 5% H_3_PO_4_) in 96-well tissue culture plates for 10 min at room temperature in the dark. The absorbance was measured at 540 nm using a microplate ELISA reader (Molecular Devices, Silicon Valley, CA, USA).

### 4.13. Statistical Analysis

All data are expressed as mean ± SEM. Data were analyzed by one-way ANOVA followed by Scheffe’s test. The statistical comparisons for two factors were made by two-way ANOVA followed by Bonferroni correction. For paired analyses, a *t*-test was used. A *p* value less than 0.05 was considered statistically significant.

## 5. Conclusions

**Figure 6:**
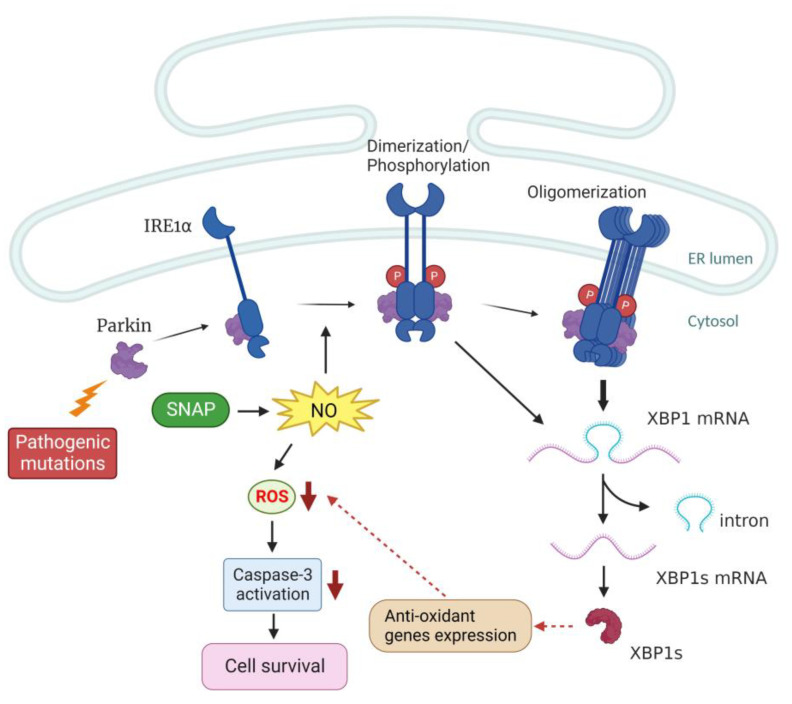
Proposed model for the functional role of parkin in preventing cell death following NO exposure. Parkin forms a complex with IRE1α under untreated conditions. After exposure to NO, parkin enhances IRE1α phosphorylation and oligomerization-mediated XBP1 splicing. Transcription of the downstream anti-oxidant genes by XBP1s [41,44,71] reduces ROS levels and caspase 3 activation, ultimately leading to protection against NO-induced cell death. However, pathogenic mutations of parkin result in impairment of this function. A dotted arrow indicates the expression of anti-oxidant genes involved in this pathway is not determined in this report. This scheme is created on http://BioRender.com (19 January 2023).

Here, we provide the first demonstration that parkin facilitates the IRE1α-triggered XBP1 splicing by augmenting IRE1α phosphorylation and oligomerization mediated probably through the parkin-IRE1α interaction, ultimately leading to amelioration of NO-induced ROS-dependent SH-SY5Y cell apoptosis. Parkin-associated pathogenic mutations impair this function (Figure 6). Because nitrosative stress contributes to the etiology of the neuroinflammation-related neurodegenerative disorders, our findings raise the possibility that the loss of these functions contributes to the pathological processes that lead to dopaminergic neurodegeneration in parkin-associated cases of genetic PD. This issue requires further investigation in primary dopaminergic neuron cultures. 

## Data Availability

The data that support the findings of this study are available from the corresponding author upon reasonable request.

## Supplementary Materials

The following supporting information can be downloaded at: https://www.mdpi.com/article/10.3390/ijms24032017/s1.
