# Supplementary figures and images for: Enhanced IRE1α Phosphorylation/Oligomerization-Triggered XBP1 Splicing Contributes to Parkin-Mediated Prevention of SH-SY5Y Cell Death under Nitrosative Stress
Source: Int J Mol Sci. 2023 Jan 19;24(3):2017. doi: 10.3390/ijms24032017 (PMC9917145; doi:10.3390/ijms24032017)

Figure S1

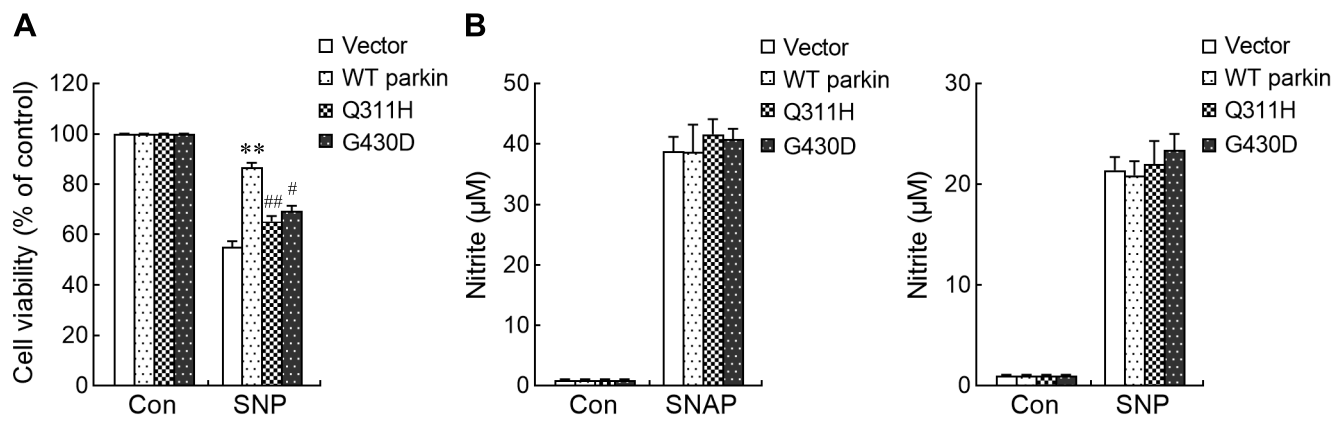

Figure S2

Fig. 1E

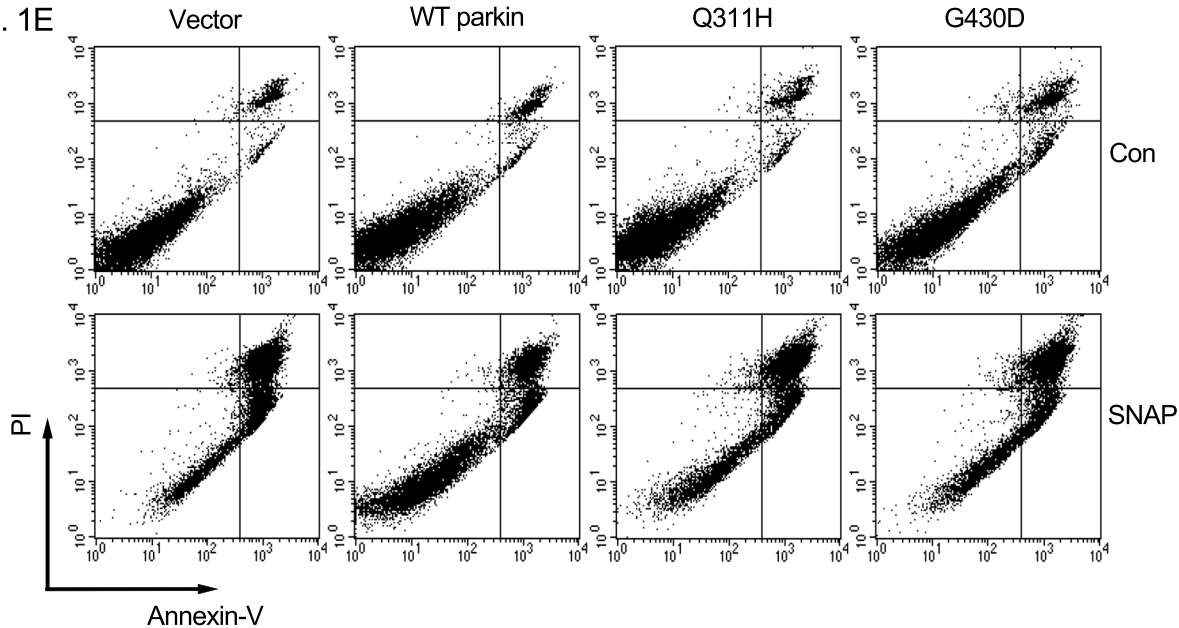

Fig. 2D

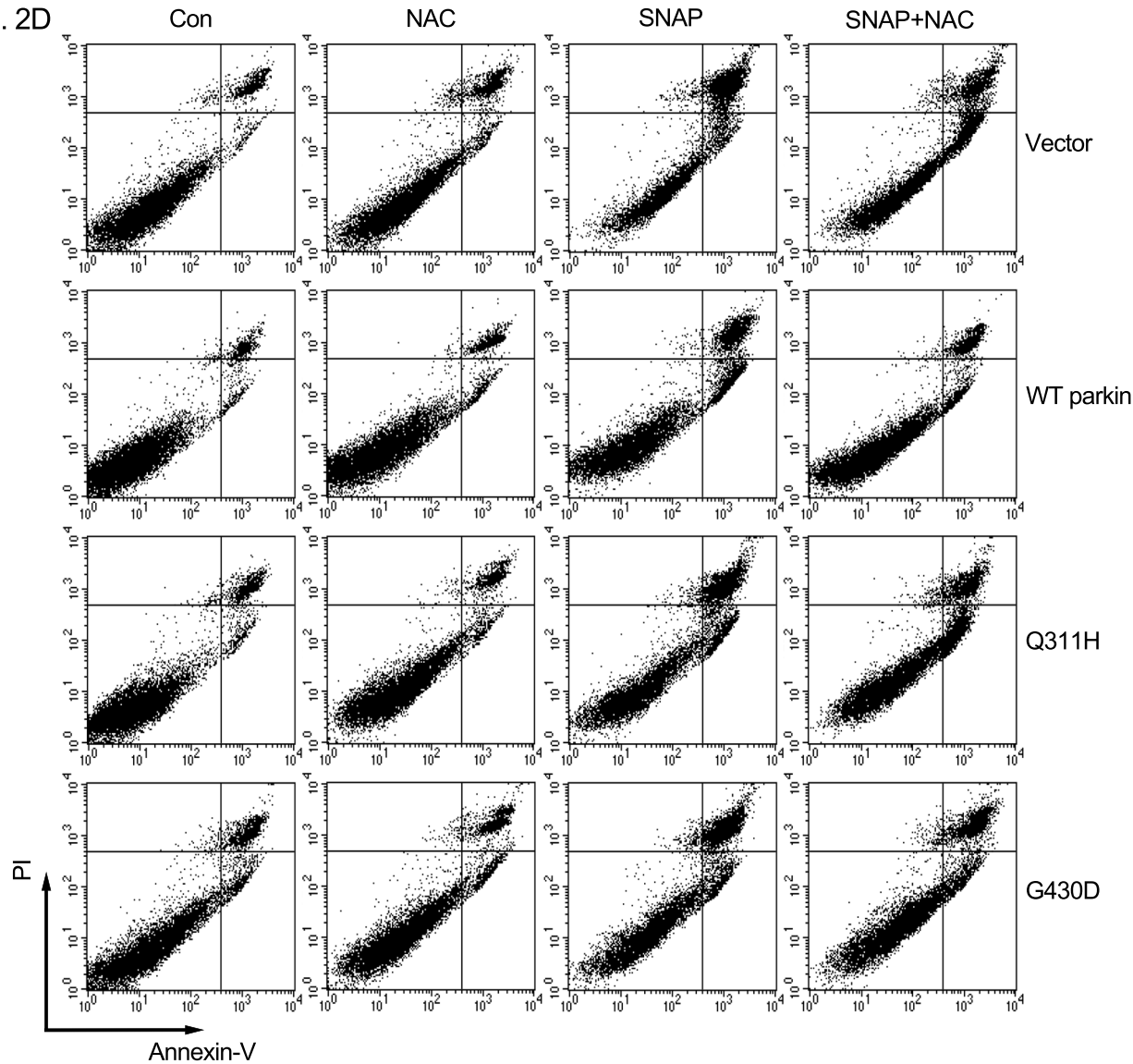

Figure S2

Fig. 4D

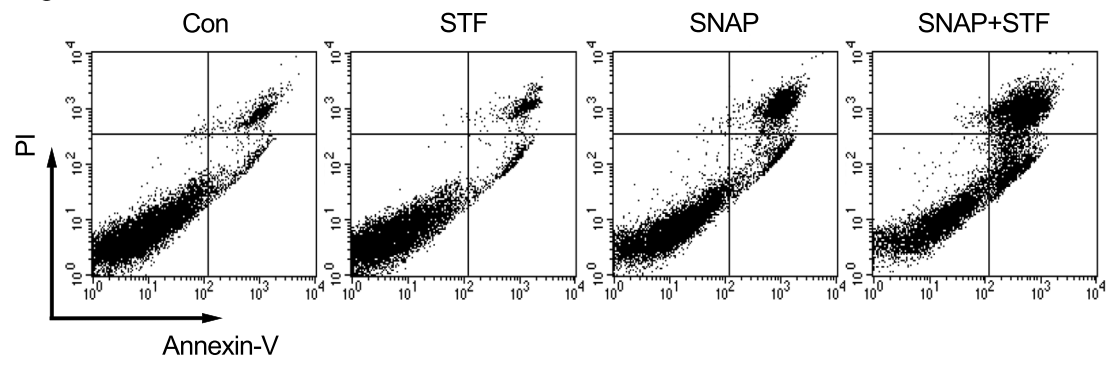

Figure S3

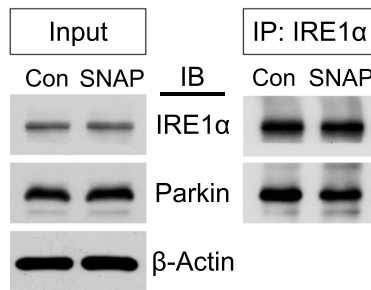

Supplement: Supplementary file 1 [file ijms-24-02017-s001.zip › ijms-2112757-supplementary.pdf]
